# Spatial patterns in sociodemographic factors explain to a large extent the prevalence of hypertension and diabetes in Aragon (Spain)

**DOI:** 10.3389/fmed.2023.1016157

**Published:** 2023-01-25

**Authors:** Carmen Bentué-Martínez, Marcos Rodrigues Mimbrero, María Zúñiga-Antón

**Affiliations:** ^1^Department of Geography and Territorial Planning, University of Zaragoza, Zaragoza, Spain; ^2^Institute of Research Into Environmental Sciences of the University of Zaragoza, Zaragoza, Spain; ^3^Health Research Institute of Aragon, Zaragoza, Spain

**Keywords:** multi-morbidity, hypertension, diabetes, social determinants of health, primary health care, decision making

## Abstract

**Introduction:**

The global burden of multi-morbidity has become a major public health challenge due to the multi stakeholder action required to its prevention and control. The Social Determinants of Health approach is the basis for the establishment of health as a cross-cutting element of public policies toward enhanced and more efficient decision making for prevention and management.

**Objective:**

To identify spatially varying relationships between the multi-morbidity of hypertension and diabetes and the sociodemographic settings (2015–2019) in Aragon (a mediterranean region of Northeastern Spain) from an ecological perspective.

**Materials and methods:**

First, we compiled data on the prevalence of hypertension, diabetes, and sociodemographic variables to build a spatial geodatabase. Then, a Principal Component Analysis (PCA) was performed to derive regression variables, i.e., aggregating prevalence rates into a multi-morbidity component (stratified by sex) and sociodemographic covariate into a reduced but meaningful number of factors. Finally, we applied Geographically Weighted Regression (GWR) and cartographic design techniques to investigate the spatial variability of the relationships between multi-morbidity and sociodemographic variables.

**Results:**

The GWR models revealed spatial explicit relationships with large heterogeneity. The sociodemographic environment participates in the explanation of the spatial behavior of multi-morbidity, reaching maximum local explained variance (R2) of 0.76 in men and 0.91 in women. The spatial gradient in the strength of the observed relationships was sharper in models addressing men’s prevalence, while women’s models attained more consistent and higher explanatory performance.

**Conclusion:**

Modeling the prevalence of chronic diseases using GWR enables to identify specific areas in which the sociodemographic environment is explicitly manifested as a driving factor of multi-morbidity. This is step forward in supporting decision making as it highlights multi-scale contexts of vulnerability, hence allowing specific action suitable to the setting to be taken.

## 1. Introduction

The coexistence of multiple chronic conditions in an individual, i.e., multi-morbidity (1), causes significant direct and indirect costs to individuals, households, and society at large (2), and can affect everyday life. The public health sector faces the challenge of mitigating the prevalence of non-communicable diseases (NCDs), a challenge that becomes increasingly threatening when multi-morbidity -the most common chronic condition today- enters the equation (3, 4). NCDs and their different modalities of multi-morbidity, are among the leading causes of premature death worldwide, being more prevalent in disadvantaged communities, thus magnifying health inequalities (5, 6).

To guide health systems in the prevention and control of NCDs, attention to the living environment and its associated risk factors is critical. The so-called Social Determinants of Health (SDH) (7) are among the most widespread frameworks that incorporate the circumstances in which people live and grow up in prevalence assessments. According to Bhattacharya et al. (8), the underlying causes of NCDs at the community level should be considered as a “collateral damage” from the interaction between SDH at different levels, including individual characteristics (genetics, age, gender, ethnicity) and choices (habits and lifestyles), living circumstances (socioeconomic status, SES), geographical settings (rural or urban environments), but also the macroeconomic and political backgrounds, along with other overarching forces.

When specifically addressing multi-morbidity, the sociodemographic structure has been highlighted as a key determinant, identifying the elderly, female, and socioeconomically deprived people as those particularly vulnerable to chronic conditions and multi-morbidity (9–12). These conditions, along with individual lifestyles and metabolic risk factors, often promote (among other conditions) hypertension and type-2 diabetes mellitus (13). These two NCDs are currently in the spotlight due to their increasing burden worldwide −422 and 1,278 million for diabetes and hypertension in 2014 and 2019, respectively (14, 15), as one of the most frequent modes of multi-morbidity (16, 17) fostered by their considerable overlap in terms of risk factors (18, 19).

There is a large body of literature devoted to multi-morbidity following a variety of approaches. For example, Johnston et al. (20) addressed the definition and measurement of multi-morbidity; Prados-Torres et al. (21) and Rajoo et al. (22) identified the most common patterns of disease association; Violán et al. (23) highlighted the importance of socioeconomic status as an aggravating factor in multi-morbidity; and Uthman et al. (13) explored the levels of vulnerability between urban and rural settings. However, the afore mentioned studies were conducted at the individual level, largely disregarding the social environment. This is also the case of the conjoint studies on hypertension and diabetes conducted in Spain. Zubeldia Lauzurica et al. (24) found that overweight and diabetes doubled the probability of developing hypertension in a population-based cross-sectional study in Valencia. Menéndez et al. (25) identified a higher prevalence of hypertension among prediabetics and diabetics in a sample of the Spanish population. The serial cross-sectional study from 2003 to 2009 conducted in several Primary Care Centers in Madrid (26) reported that patients with type 2 diabetes who also suffered from hypertension increased from 89.78% in 2003 to 94.76% in 2009, this percentage being higher for women and for patients older than 65 years. Urrutia et al. (27) in a cohort study on risk factors for diabetes in the Basque Country found associations in the prevalence of hypertension, obesity, family history of diabetes and low educational level. Ramón-Arbués et al. (28) identified significant associations between overweight and obesity and the prevalence of diabetes, dyslipidemia, hypertension, and metabolic syndrome in a sample of 23.729 workers in Aragon.

This study offers an ecological perspective that adds to what is known by incorporating the spatial perspective. We analyze the influence of the social and demographic spheres of the SDH on hypertension-diabetes multi-morbidity at the community level under the premise that disadvantaged communities are more likely to attain a higher prevalence of both diseases, although this link is not necessarily equally strong everywhere. Therefore, our objectives were to (i) determine the extent to which spatial patterns of hypertension and diabetes prevalence overlap (referred to as “multi-morbidity”), (ii) determine whether these spatial patterns are related to the sociodemographic factors that explain multi-morbidity and, if so, (iii) investigate possible spatially varying relationships between SDH and multi-morbidity. The methodological framework leverages the use of Geographic Information Systems (GIS) and spatial analysis techniques that have proven to be very useful in the field of epidemiology (29–31). First, we applied Principal Component Analysis to determine the spatial patterns of multi-morbidity and synthesized the main sociodemographic factors into a meaningful indicator. Then, we applied Geographically Weighted Regression [GWR; Fotheringham et al. (32)] to ascertain whether the association between variables shows spatial nuances. In line with the Health in All Policies (HiAP) approach (33), this broader perspective offered by spatial analysis in health research advances beyond individual factors (34) being particularly useful to support decision making outside the direct remit of the health sector (e.g., territorial planning and health-related policy formulation).

## 2. Materials and methods

### 2.1. Regression data

#### 2.1.1. Prevalence of hypertension and diabetes

**Figure 1:**
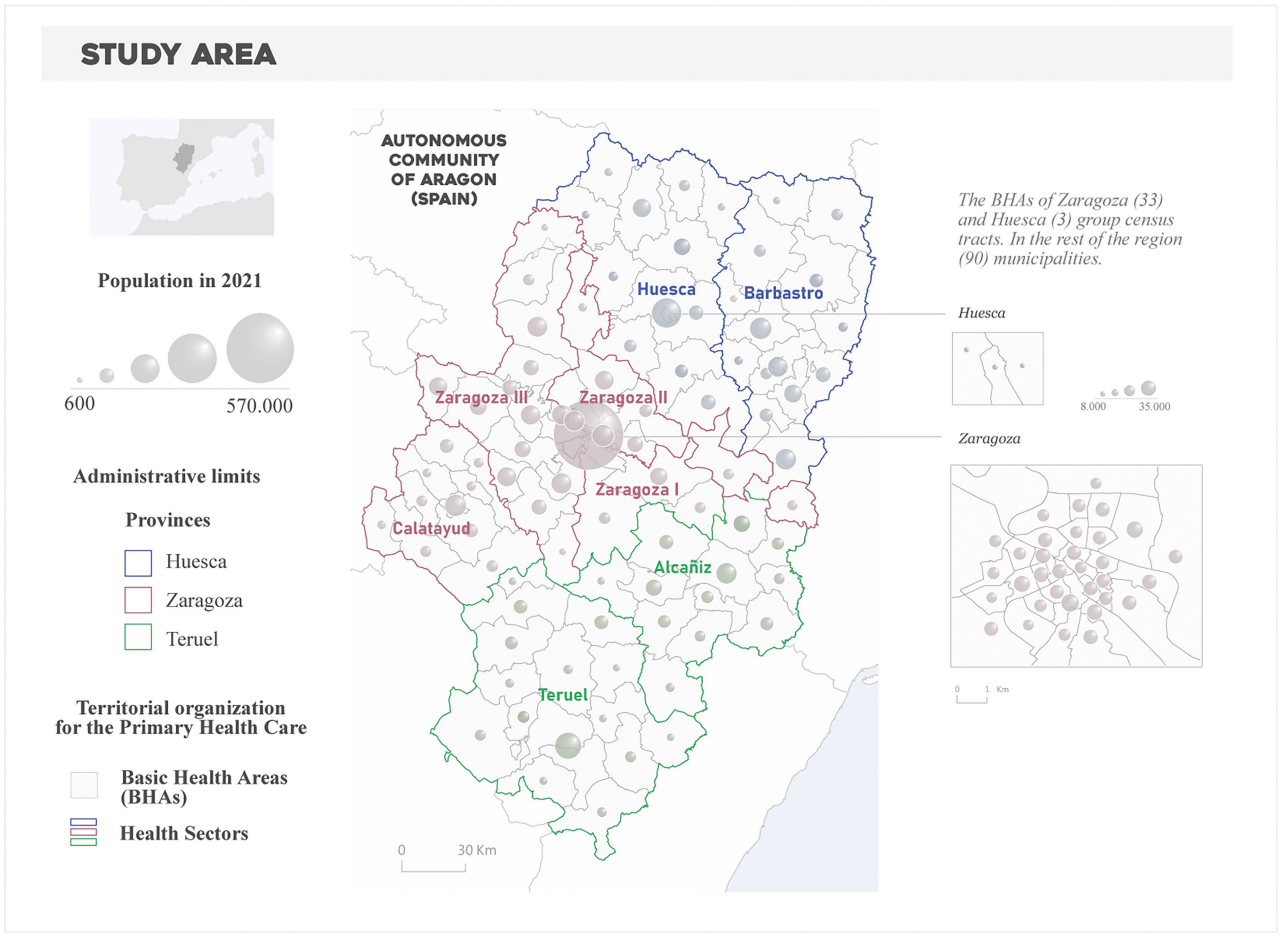
Study area. Territorial organization of the Primary Health Care in Aragon.

Data on prevalence of hypertension and diabetes are freely available in the Aragon Health Atlas.^1^ The measure of each morbidity dimension refers to the entire population (children and adults) with an active diagnosis in hospital and/or primary care information systems, which offers the total number of diagnosed cases by age class. Hence, we analyze the number of cases by sex and age, exploring separate annual records in 2015, 2016, 2017, 2018, and 2019. Raw figures were standardized by sex and age to override potential bias among age or gender-related group classes, enabling their comparison. The study area is the Autonomous Community of Aragon, a northeastern region of Spain (Figure 1). Aragon is a paradigmatic example of dissimilar population systems. It encompasses a major metropolitan area gathering half the population (Zaragoza), while low densely populated and rural settlements occupy the rest of the region (47.720 km^2^). The spatial unit of analysis at which prevalence data were retrieved was the Basic Health Area (BHA) level, the territorial and administrative unit for Primary Health Care in Aragon. The BHA was specifically designed to portray homogenous regions in terms of population and health-related services. Aragon is organized in 123 BHAs corresponding to 8 Health Sectors [Huesca, Barbastro (province of Huesca), Zaragoza I, Zaragoza II, Zaragoza III (province of Zaragoza], Teruel and Alcañiz (province of Teruel).

#### 2.1.2. Social determinants of health indicators

Social determinants of health data were provided (and free available) by the Municipal Register of Inhabitants, the Atlas of Household Income Distribution, and the Census of Population and Housing offered by the Spanish Statistics Institute.^2^ We retrieved information about four SDH indicators about demographic characteristics and socioeconomic status -income and education-) at census tract level, being subsequently aggregated into the BHA structure to match prevalence data. These four indicators refer to key driving factors of multi-morbidity as indicated in the literature (10). As variables in the demographic’s domain, we retrieved the percent population of 65 years and older and the percent population of 85 years and older, both obtained from the Municipal Register of Inhabitants as per 2015. In the case of the socioeconomic status, we considered the average household income from the Atlas of Household Income Distribution. As is explained in the Atlas’ methodological guide, “*Income per consumption unit is obtained*, *for each household*, *by dividing the total net household income by the number of consumption units*. *The distribution of persons is taken*.” Income is expressed in € and the reference date is 2015. Education attainment was measured using the average education level, depicted as the sum of class marks per level of studies of people aged 16 and over, divided by the total number of people of those ages by census tract. It is expressed as a mean-continuum value between 1 and 4 corresponding to (1) Without studies, (2) First grade, (3) Second grade, and (4) Third grade. The source is the Population and Housing Census of 2011.

### 2.2. Methods

The methodological procedure was developed in two steps. First, we applied PCA to the original dataset to generate specifically devoted indicators of multi-morbidity and the sociodemographic environment. These indicators will subsequently act as response (multi-morbidity) and explanatory variables (sociodemographic environment) in regression analysis. Then, we used GWR to examine the strength of the relationship between multi-morbidity and the sociodemographic environment, paying special attention to the underlying spatial patterns of performance and association. The software used for the implementation of the analyses was RStudio in the case of PCA, GWR4.0 in the case of GWR and ArcMap for the generation of the cartography.

#### 2.2.1. Principal component analysis

The PCA consists of a linear transformation through linear combinations of a set of original variables to produce a new array of independent dimensions, known as principal components. The new configuration of dimensions synthesizes the original information, each component capturing a fraction of the original variance in decreasing order of importance. The usefulness of PCA in this study was twofold. On the one hand, it allows us to derive a multi-morbidity indicator from the data on prevalences of hypertension and diabetes. The resulting components captured the joint variation in both prevalences, which in this work we referred to as multi-morbidity. On the other hand, new sociodemographic indicators are highly prone to collinearity, impairing certain types of regression like ordinary least squares (OLS) and, consequently, GWR (which was originally devised as a spatial disaggregation of OLS). Hence submitting the selected variables to PCA solves the collinearity among the explanatory factors of the regression models that was detected during a previous explanatory analysis.

To achieve a comprehensive analysis of multi-morbidity, we constructed multiple realizations of the indicator (*via* PCA). In all cases, prevalence data were analyzed by sex, exploring both annual ├indicators, that is, an individual multi-morbidity indicator per year (and sex), and an aggregated indicator comprising data during the entire study period. This led to 12 multi-morbidity indicators that were further investigated using GWR. In turn, the sociodemographic indicators were submitted to a single PCA. The selection of the key components (either multi-morbidity or socioeconomic) was based on the Kaiser criteria, i.e., retaining only those components with an eigenvalue greater than one (35). The resulting components were subsequently submitted to correlation analysis (Pearson’s R) to ascertain the potential association between multi-morbidity and sociodemographic environment.

#### 2.2.2. Geographically weighted regression

Geographically weighted regression techniques extend the traditional use of regression models (in this context known as global regression models), through the assessment of local regression parameters (32).

In GWR, a region is described around each observation location *i* (with coordinates *ui*,*vi*) and all the data points within a given neighborhood window are used to calibrate a regression model attributed to that location. This process is repeated over all the candidate locations obtaining a set of local regression statistics as a result. GWR applies a distance-based weight pattern; hence, observations closer to the center of the window are weighted more heavily. The following equation describes a conventional GWR mathematically:


yi=∑kβk(ui,vi)xk.i+εi

where *yi*, *xk*,*i* and *εi* are, respectively, a dependent variable, *kth* an independent variable, and the Gaussian error at location *i; (ui*,*vi)* is the x-y coordinate of the *ith* location; and coefficients *β (ui*,*vi)* vary.

When determining the size of the neighborhood region (bandwidth calibration) two strategies can be followed: (i) fixed kernel, which specifies a distance threshold equally wide for each regression point location, thus considering a variable number of points in each local regression estimate (i.e., the central location plus those neighboring observations that fell within the window); and (ii) adaptive kernel, which permits a larger or small bandwidth when the data are sparse or densely distributed, adapting the bandwidth to a predetermined number of observations required to fit the regression. The first is generally more adequate when observations are homogeneously distributed over space and the latter when there is evidence of clustered patterns. In our study area the BHA distribution is highly skewed, with a rather homogeneous distribution and size of most BHAs (90), except those in the main urban settlements (the cities of Zaragoza and Huesca; 33), difficulting the *a priori* selection of a weighting scheme. Accordingly, both strategies were compared. The selection of the bandwidth size was optimized by minimizing the Akaike Information Criterion (AIC), adapted for GWR by Hurvich et al. (36).

The outcomes from a GWR model include the usual parameter estimates of an OLS regression (beta coefficients, standard error, and coefficient of determination) but grants an individual set per each location. This allowed us to address the spatial pattern of the relationships (beta coefficients and standard error) and the percent of variance captured by the model (R^2^). Likewise, model inputs must fulfill the usual requirements of homoscedasticity, independence, or normality of the covariates (which were all satisfied). In this research, we employed the GWR 4.09 software.^3^ A set of GWR explanatory models were fitted for male and female multi-morbidity –all years and each year-, separately. For each model we mapped the regression coefficients and the percent of variance explained (local R^2^).

## 3. Results

### 3.1. Multi-morbidity and sociodemographic patterns

According to the Kaiser criteria, only the first component of all PCA models -both multi-morbidity and SDH- was meaningful and was thus retained for further analysis. The proportion of the variance explained by the first components of multi-morbidity ranged from 89 to 91% in male models to 93–94% in female models (Table 1A). The loadings of both prevalence rates in the first component stood consistently lower than −0.93 in all models, meaning that those BHAs with negative scores depicted a large overlap of high prevalence rates, i.e., increased multi-morbidity. The first component of the SDH model -the sociodemographic environment-, attained a 74% of variance (Table 1B). The loadings of the overaging indicators -population older than 65 and 85- were high and negative (−0.88 and − 0.90, respectively), as opposed to those of educational level (0.87) and average household income (0.76). Thus, negative values correspond to an overaged population structure with socioeconomic disadvantages, in contrast to the better socioeconomic situation -higher level of education and income- that the positive values indicated.

**Table 1:** Description of the selected PCs.

(a) Multi-morbidity of hypertension and diabetes PC							
Male				Female			
PC1	%V	Variables*	EigVal	PC	%V	Variables*	EigVal
All years	0.89	H15	−0.94	All years	0.92	H15	−0.96
		H16	−0.93			H16	−0.96
		H17	−0.94			H17	−0.97
		H18	−0.95			H18	−0.97
		H19	−0.95			H19	−0.96
		D15	−0.94			D15	−0.95
		D16	−0.94			D16	−0.96
		D17	−0.94			D17	−0.95
		D18	−0.94			D18	−0.94
		D19	−0.94			D19	−0.95
2015	0.9	H15	−0.94	2015	0.94	H15	−0.97
		D15	−0.94			D15	−0.97
2016	0.89	H16	−0.95	2016	0.93	H16	−0.97
		D16	−0.95			D16	−0.97
2017	0.89	H17	−0.95	2017	0.93	H17	−0.96
		D17	−0.95			D17	−0.96
2018	0.9	H18	−0.95	2018	0.93	H18	−0.96
		D18	−0.95			D18	−0.96
2019	0.91	H19	−0.95	2019	0.94	H19	−0.97
		D19	−0.95			D19	−0.97
(b) Sociodemographic environment PC							
PC1	• %V	• Variables*	• EigVal	*P65: Population over 65 years			
				*P85: Population over 85 years			
				*EDU: Education attainment			
	0.74	P65	−0.88	*INC: Average household income			
		P85	−0.9				
		EDU	0.87				
		INC	0.76				

EigVal denotes the correlation value between the original variables and the PCs. *H, hypertension; D, diabetes. %V indicates the proportion of variance within the block gathered by the PC.

**Figure 2:**
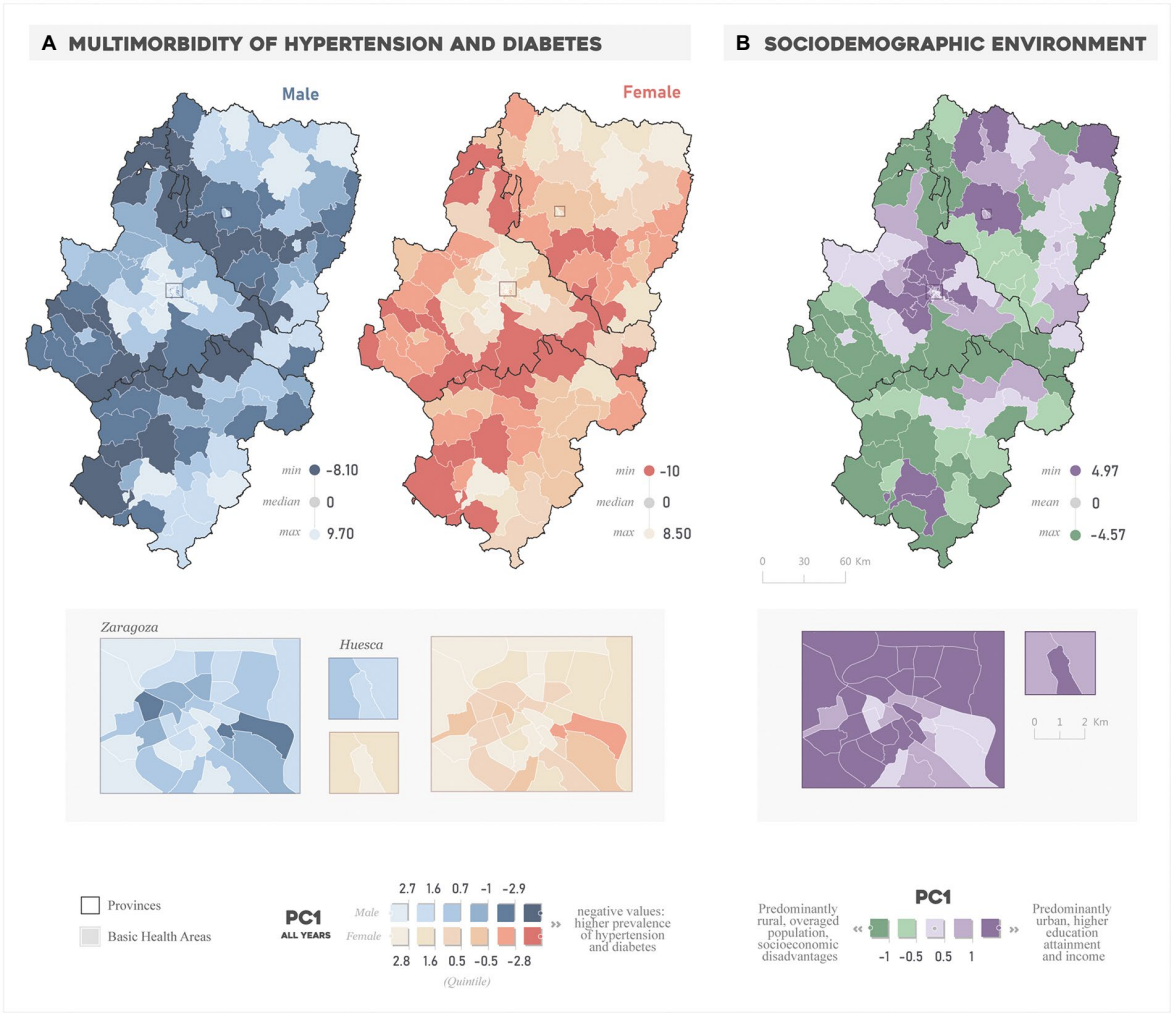
Spatial distribution of the **(A)** “All years” multi-morbidity PC and the **(B)** sociodemographic environment PC.

The most vulnerable BHAs in terms of multi-morbidity during the entire study period (Figure 2A) were located along the borders of the province of Zaragoza, as well as in the southeastern fringe (similar patterns were observed in the annual multi-morbidity, see Supplementary Figure S1). In these areas, the prevalence of hypertension in men ranges between 20 and 30% and in women between 25 and 35% **(**Supplementary Figure S2). In the case of diabetes, the rates oscillate between 8 and 10% in both sexes (Supplementary Figure S3). The urban BHAs (Zaragoza and Huesca) show a lower prevalence and multi-morbidity values. However, BHAs with greater vulnerability appear in the central-eastern and southwestern sector of the city of Zaragoza, with prevalence rates around the average of the entire period, 20.8 and 23.2% in the case of hypertension and 8.5 and 6.8 for diabetes -men and women, respectively. On the other hand, the BHAs in the northeast and southeast of the provinces of Huesca and Teruel, respectively, and in the metropolitan area of the city of Zaragoza have positive multi-morbidity PC values (lower overlap), also corresponding to prevalence rates in the lowest quintiles.

Regarding the sociodemographic environment (Figure 2B), the BHAs with the highest values correspond to the areas that lead the functional hierarchy of settlements in Aragon: the provincial capitals of Zaragoza, Huesca, and Teruel and the BHAs in the northwest of the province of Zaragoza and north of Huesca. These are characterized by a younger demographic structure and a higher education attainment and income compared to those BHAs with the lowest scores. The latter have a distinct rural character also featuring a lower socioeconomic level and a highly overaged population.

#### 3.1.1. Spatial variation in the association between variables

We observed a strong global link between hypertension-diabetes and the sociodemographic environment (Supplementary Figure S4). The correlation was higher in the prevalence of hypertension (average of 0.75 in male; 0.87 in female) than that of diabetes (0.67; 0.81, male and female, respectively). The direction of the associations is negative, meaning an increase in prevalence values parallel to the decrease in the sociodemographic PC value -i.e., elderly population, less education attainment and income-. In an opposite direction -but as previously noted, the same meaning-, the correlation with the multi-morbidity PCs was lower in male than in the female ones (average of 0.73 and 0.87, respectively) but keeping similar correlation rates that both prevalence separately.

From a spatially-explicit standpoint, the GWR models outperformed the global OLS (Table 2). Overall, the fixed kernel bandwidth strategy offered a better fit compared to the adaptive one, hence the fixed GWR model was selected in this study. The number of neighbors (bandwidth size) (optimized by minimizing the AIC) resulted similarly for both male and female models: 30 and 27 km (similar results in 2015–2019 models, see Supplementary Figure S6). The difference between GWR and OLS in terms of percent of variance explained (R2) stood at 0.20 and 0.13 in male and female models, respectively. This suggests that, despite a solid global relationship do exist, the spatial disaggregation of such models does a better job in capturing the conjoint variance of prevalence. As already evidenced by correlation analysis, female models performed better, with R2 values exceeding 0.85 in all years 0.75 in the male models. In both cases, the percentage of variance explained is higher at the beginning (2015) than at the end (2019) of the period studied, though by a slim margin. It follows from these results that the associations between multi-morbidity and sociodemographic environment vary spatially.

**Table 2:** Percent of variance explained (R^2^) by regression models.

R2	OLS	GWR		OLS	GWR	
		Fixed	Adaptive		Fixed	Adaptive
M_ALL_	0.57	0.76	0.61	0.77	0.87	0.78
M15	0.6	0.76	0.64	0.79	0.91	0.8
M16	0.58	0.74	0.62	0.78	0.87	0.79
M17	0.54	0.74	0.58	0.73	0.85	0.75
M18	0.56	0.74	0.6	0.73	0.85	0.75
M19	0.55	0.73	0.58	0.72	0.85	0.75
	Male			Female		

**Figure 3:**
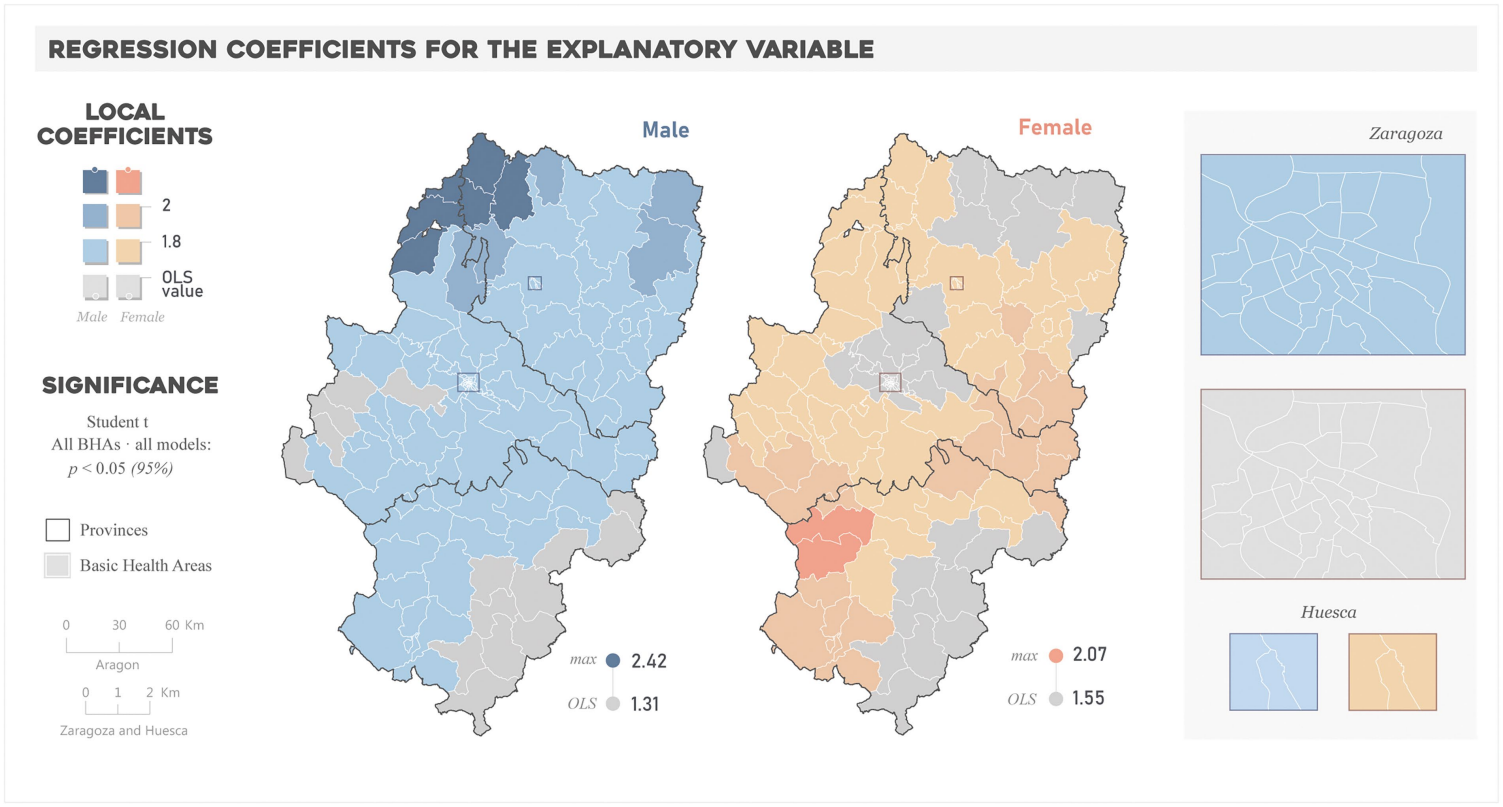
Spatial distribution of the regression coefficients for the explanatory variable in “All years” models.

Figure 3 shows the spatial patterns in regression coefficient values in “All years” fixed GWR models (again, similar patterns were found in yearly models, see Supplementary Figure S5). All beta regression coefficients were found significant (*p* < 0.05) and positive. The positive values indicate a direct relationship, hence the more the advantageous the situation (younger population with higher income and educational attainment) the lower the rate of multi-morbidity of hypertension and diabetes. But this behavior was not equally strong over the study region and showed manifold spatial nuances.

The comparison between local and global coefficients enables determining spatial differences in the relationships, i.e., where the change in prevalence relative to the socioeconomic environment is more pronounced (GWR > OLS) or smoother (OLS > GWR) than that globally estimated. In the case of male models, the magnitude of local coefficients was equal or higher than the OLS (1.31) in most of BHAs, reaching the highest values (up to 2.42) in the BHAs in the northwestern part of the region. In the case of female models, fewer BHAs exceeded the OLS coefficient (1.55), and the higher values (up to 2.07) correspond to those of the middle east and southwest of the region.

**Figure 4:**
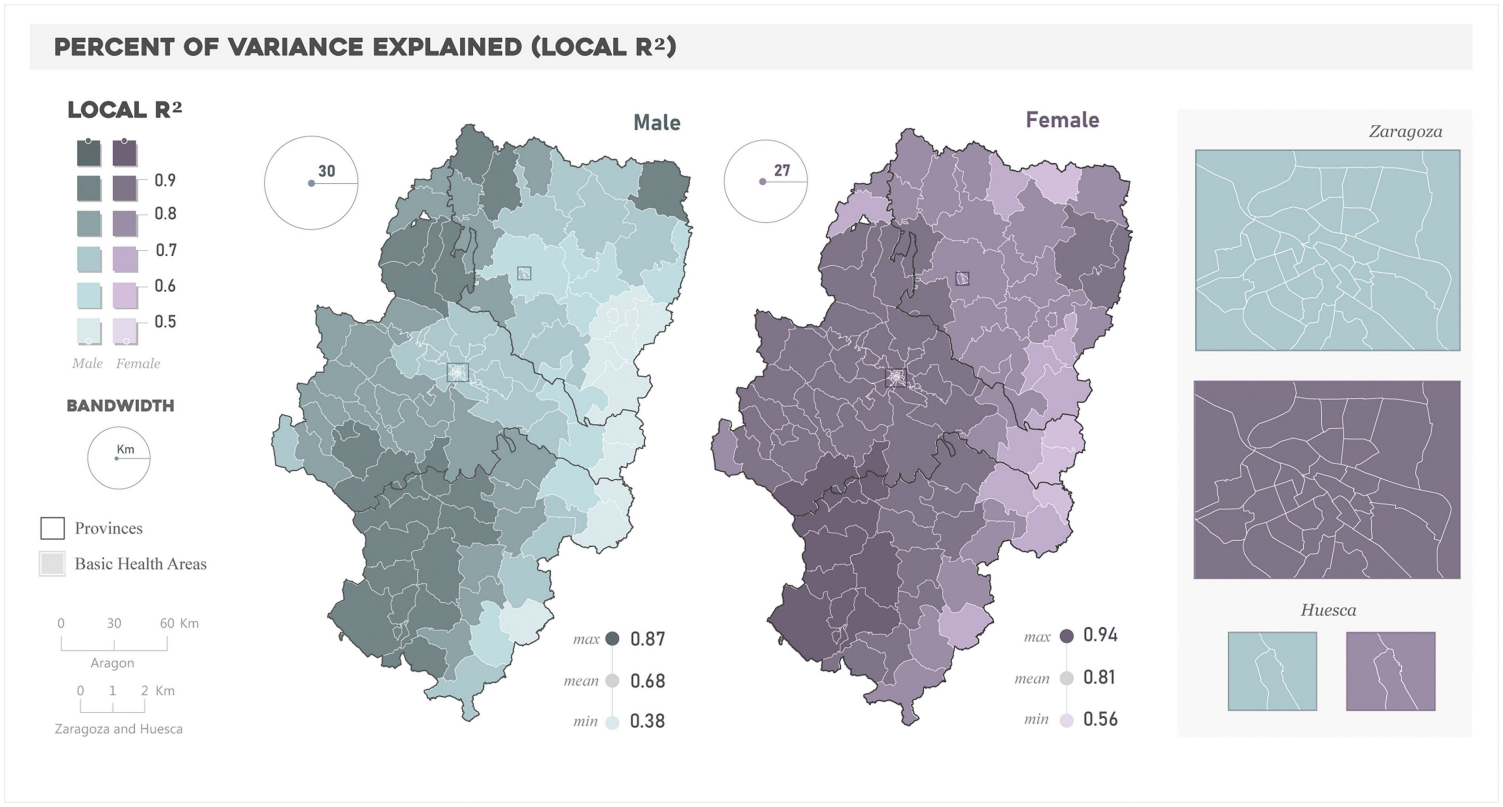
Spatial distribution of the Local R2 in the “All years” models.

Differences were also noteworthy in terms of model performance (Figure 4 and Supplementary Figure S6). The percent of the variance explained by the GWR model ranged from 0.38 to 0.87 and 0.56 and 0.94 in male and female models, respectively. The highest R^2^ values in male models clustered in the northwestern and southwestern ends of the region, with the lowest values located in the eastern areas. This west–east gradient also appeared in female models, but R2 values exceeded 0.7 almost over the whole study area.

## 4. Discussion

The aim of this study was to investigate the spatial behavior of the overlapping prevalence of hypertension and diabetes (“referred to as multi-morbidity”) from an ecological perspective. We leveraged a combination of PCA and GWR techniques to generate variables related to multi-morbidity by sex in different years and sociodemographic factors; and analyze whether the relationships between variables (PCs) were stationary across the study area. Our results allowed us to accept the working hypothesis, safely concluding that sociodemographically disadvantaged territories were more likely to suffer a higher burden of multi-morbidity, though this relationship does not necessarily hold elsewhere.

The prevalence rates of hypertension were, as an average for the period studied (2015–2019), of 20.8 and 23.2% for hypertension and 8.5 and 6.8% for diabetes, male and female, respectively. In the case of hypertension, the percentage exceeds the average for Spain (15.7% -male- and 17.3% -female-), while in diabetes the figures were similar (7.3% male and 6.1% female) (37). In terms of spatial variability, the percentage differences between autonomous communities in Spain are up to 12% in hypertension and 4% for diabetes. The spatial variability observed in Aragón is much higher, with percent differences of up to 25 and 12% for hypertension and diabetes in men, and up to 35 and 13% in women.

The results indicate that the spatial patterns of population aging, educational level, and income distribution explain the multi-morbidity patterns of hypertension and diabetes to a large extent (average R2 0.75 and 0.87 in male and female models, respectively). These findings are in line with the literature. In the study of Chidumwa et al. (38) about shared risk factors of hypertension and diabetes, the elderly, female and people with lower education and household wealth were associated with higher prevalence. Tirapani and Fernandes (39) concluded on the importance of factors such as income and education impact on the prevalence, incidence, diagnosis, treatment, progression, and mortality of hypertension and diabetes mellitus. Pandey et al. (40) also found that age and level of education seemed to be associated with comorbid diabetes and hypertension. In Spain, Bennett et al. (41) estimated incidence trends by deprivation for cardiovascular disease, hypertension and type 2 diabetes and found higher incidence levels in the most deprived areas. Zghebi et al. (42) found a higher vulnerability of comorbidity of diabetes in women, the elderly and the most deprived.

But the GWR analysis emphasized the non-stationary behavior in terms of explanatory power and influence of the sociodemographic factors. Previous studies modeling the relationships between these diseases and socioeconomic risk factors through GWR techniques have also found regional variations. Ogunsakin (43) revealed that educational status had different effects across provinces of South Africa when investigating diabetes and hypertension. Park et al. (44) addressed the hypertensive disease mortality in South Korea and found that the effects of socioeconomic status were only consistent in some regions of the country. Hipp and Chalise (45) and Quiñones et al. (46) found that indicators such as poverty were associated with diabetes in some regions in the United States. In Europe, Kauhl et al. (47) found spatially varying strength of the association between demographic variables and hypertension, and concluded that persons aging in deprived areas in Northeastern Germany will be at greater risk of hypertension. These authors also found that the strength of the association between diabetes mellitus and socio-demographic variables such as age and employment conditions displayed strong regional variations (48). The spatially-varying nature of the relationships has implications beyond the mere variability, since different intensities of the relationship relate to higher influence of the socioeconomic environment; therefore, more susceptible to be the priority target of public health policies and regulations. In this sense, we observed that the strength of the observed relationships was sharper in men’s prevalence, while women’s models attained more spatially homogeneous and with higher explanatory performance. From this we may deduce that if sociodemographic disadvantages increased in Aragonese regions, the prevalence of multi-morbidity of hypertension and diabetes in women would be more likely to increase in a wider part of the autonomous community, while in certain regions men’s prevalence would be more prone. In the case of men, the vulnerability would be high in northwestern areas. Although these results deserve to be contrasted with individual-level studies concerning differences between sexes, they are in line with the statements of the Spanish Ministry of Health in the Clinical Indicators in Primary Care in Spain report: the social gradient in the prevalence of hypertension and diabetes is more pronounced in women (37).

However, several considerations need to be addressed to properly contextualize our findings. This study stems from data sources with differences in terms of spatial aggregation and time frame. For example, the demographic structure and income indicators were reasonably up-to-date but information on the educational level comes from the last Spanish Population Census published in 2011. Likewise, in studies such as this one, in which the territorial organization of the health system is based on areas grouping several municipalities in some cases, and segregating them into census tracts in others, the broad change in scale may hinder the relationships, though the GWR approach overcomes that limitation to some extent. But devising specific, methodological protocols for urban BHAs, for which more detailed sources of information (e.g., neighborhood, block, or building) are often available, would be preferable. The research by de Cos Guerra (49) on methodological alternatives and sources of information to study residential vulnerability at the intraurban level exemplifies this line of reflection. It would also be of interest to take advantage of European initiatives such as Urban Audit (50) to generate databases and develop comparative studies between European cities.

Another consideration to be accounted for was the absence of other spheres of HD in our analysis. Hypertension and diabetes, and in general terms most chronic conditions, are associated with unhealthy and irregular dietary and sleeping patterns, stressful lifestyles, sedentarism and alcohol and tobacco consumption (51, 52). However, this information is not generally available at the population-level and therefore can hardly be incorporated in ecological studies. In this context we believe it is relevant to combine the results of studies conducted at both individual and population level. In Aragon, the opportunity would come from the EpiChron Cohort of Prados-Torres et al. (53), a database that integrates clinical, sociodemographic, and medical practice information of all inhabitants registered as users of the public health system in Aragon since 2011. Likewise, it would be interesting to incorporate other diseases into the multi-morbidity pattern analysis (e.g., obesity), as they have already been conducted in Aragon by Abad-Díez et al. (54) and Ramón-Arbués et al. (28).

Future lines of development shall focus on incorporating indicators related to the organizational structure of the health system, paying attention to the availability of diagnostic resources and the Variations in Medical Practice (VMP). The behavior of these factors could condition the existence of situations of under-diagnosis of these diseases, which in fact has already been observed in previous studies in Spain. Catalá-López et al. (55) published a systematic review and meta-analysis about the control of arterial hypertension in Spain and concluded that the control of hypertension is far from optimum and that patients at risk with comorbidities appear to be controlled worse. de Burgos-Lunar et al. (26) estimated in their study population a 30% of patients with type 2 diabetes suffering previously undiagnosed hypertension in 2003, and 23.1% in 2009. In the case of Menéndez et al. (25), undiagnosed hypertension was identified in 37.4% of patients. Tamayo-Marco et al. (56) reported a proportion of unknown cases of diabetes nearly 50% in their Aragonese sample population. It is also worth mentioning that, in the specific case of Aragon, there has also been talk of under-diagnosis of other NCDs such as Chronic Obstructive Pulmonary Disease (57) and depression (58). Regarding the VMP, in Spain there are previous initiatives such as the Atlas of VMP in the Spanish National Health System (59). One of the lines of work of this atlas has translated in the Atlas of diabetes health care in Aragon, in which vulnerability profiles of diabetes by sex, age (from 40 years and older) and SES are showed. Indeed, spatial patterns of such vulnerability by SES have been explored, and only in some cases do they correspond to those identified in this work. This could be because the age group to which both studies refer is different. Therefore, it is worth reconsidering for the future whether the associations between prevalence, multi-morbidity and HD vary when stratifying by age groups.

Finally, with respect to spatial analysis techniques, this work has proposed a methodological itinerary based on PCA and GWR that has allowed us to achieve the proposed objectives. However, it is considered relevant to enlarge and contrast the results of the multi-morbidity patterns obtained by PCA with those offered by other spatial techniques such as cluster identification or hot-spot analysis (34, 60). Similarly, it is proposed to extend the time horizon of study to carry out multi-temporal analyses and deepen the scale changes by using the Multi-scale GWR (MGWR) that allows to differentiate local, regional and global processes (61).

## 5. Conclusion

The prevalence of hypertension and diabetes in Aragon displays a large covariation in a large part of the territory. This spatial overlap, which in this work we have referred to as multi-morbidity, is higher in territories with a disadvantaged sociodemographic environment. Factors such as aging, salary and level of education present local patterns in their capacity to explain prevalence, meaning the existence of particularly vulnerable areas. These results are put at the service of decision making to guide the prioritization of interventions aimed to manage multi-morbidity and health inequalities with which it is associated. The integration of spatial data on diseases and SDH in a GIS tool makes it possible to identify health inequalities related to different indicators. By combining this GIS support with GWR modeling it is possible to predict how the disease-SDH behavior would be when intervening on a specific SDH dimension. Moreover, making conditional queries would make it possible to cross-reference health outcomes with variables related, for example, to the organization of health care services. As a result of these queries, those areas that meet certain criteria, such as a higher prevalence of diseases and unfavorable situations in terms of accessibility to equipment and services, could be considered as priorities for intervention -allocation of resources- to favor preventive, diagnostic and treatment capacity. Finally, it is worth mentioning that the methodology of this work could be replicated with other chronic diseases, as well as in other Spanish regions or even in other countries.

## Data availability statement

The datasets presented in this study can be found in online repositories. The names of the repository/repositories and accession number(s) can be found in the article/Supplementary material.

## Ethics statement

The studies involving human participants were reviewed and approved by Research Ethics Committee of the Autonomous Community of Aragon: CEICA. Written informed consent for participation was not required for this study in accordance with the national legislation and the institutional requirements.

## Author contributions

CB-M: conceptualization, methodology, investigation, data curation, writing-original draft, and visualization. MM: conceptualization, methodology, investigation, software, validation, formal analysis, writing-review and editing, and supervision. MZ-A: conceptualization, methodology, investigation, resources, writing-review and editing, supervision, project administration, funding acquisition, and funding. All authors contributed to the article and approved the submitted version.

## Funding

This project was partially funded by the Group of Studies in Territorial Planning financed in the call for research groups of the Government of Aragon (BOA, Num 62, March 26, 2020) and Government of Aragon through the Predoctoral Fellowship granted to CB-M.

## Conflict of interest

The authors declare that the research was conducted in the absence of any commercial or financial relationships that could be construed as a potential conflict of interest.

## Publisher’s note

All claims expressed in this article are solely those of the authors and do not necessarily represent those of their affiliated organizations, or those of the publisher, the editors and the reviewers. Any product that may be evaluated in this article, or claim that may be made by its manufacturer, is not guaranteed or endorsed by the publisher.

## Footnotes

^1^https://idearagon.aragon.es/atlas/Aragon/info/salud-publica

^2^https://ine.es/

^3^https://gwr.maynoothuniversity.ie/gwr4-software/
