# Supplementary figures and images for: Spatial patterns in sociodemographic factors explain to a large extent the prevalence of hypertension and diabetes in Aragon (Spain)
Source: Front Med (Lausanne). 2023 Jan 25;10:1016157. doi: 10.3389/fmed.2023.1016157 (PMC9905822; doi:10.3389/fmed.2023.1016157)

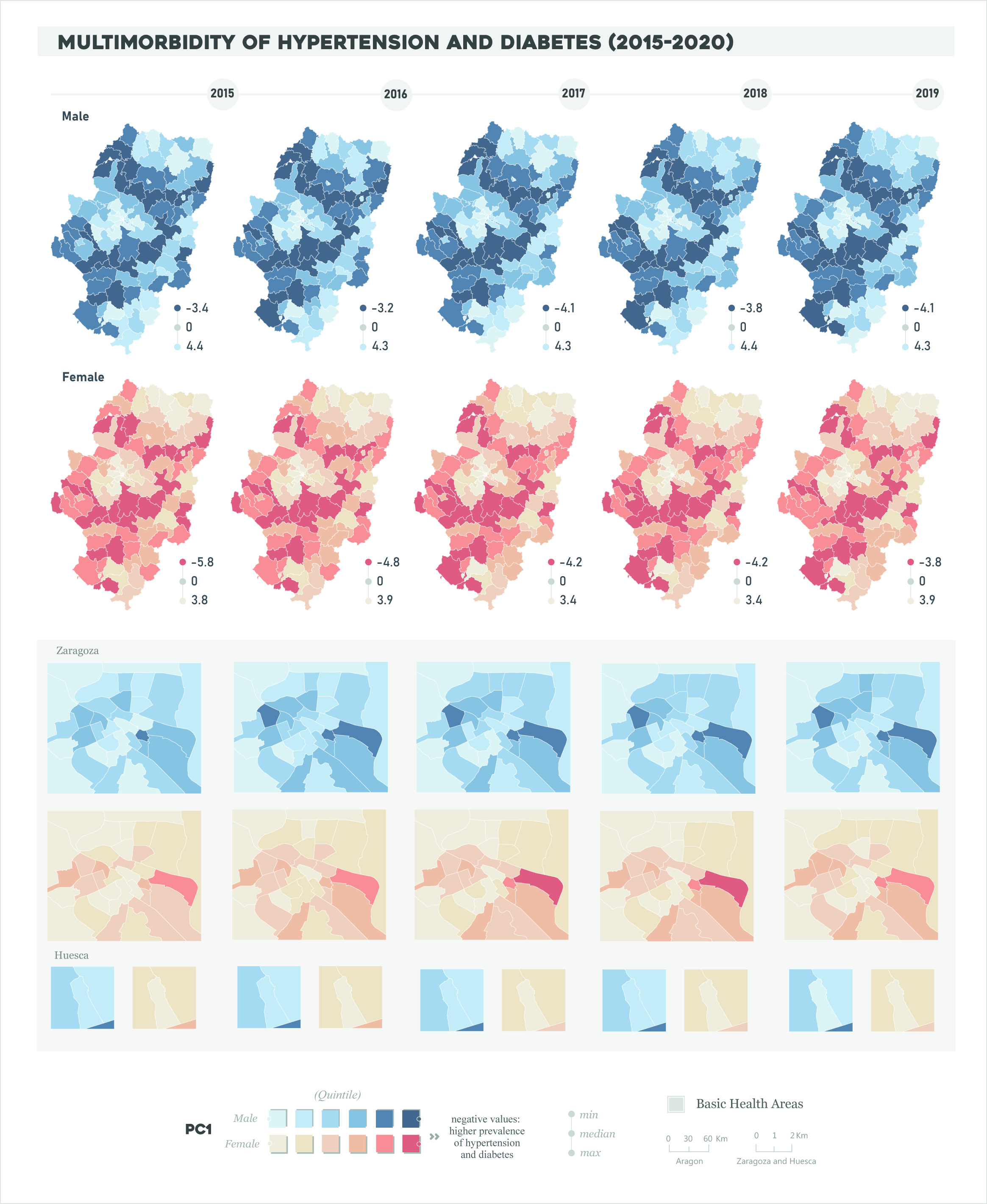

Supplement: Supplementary file 1 [file Image_1.JPEG]

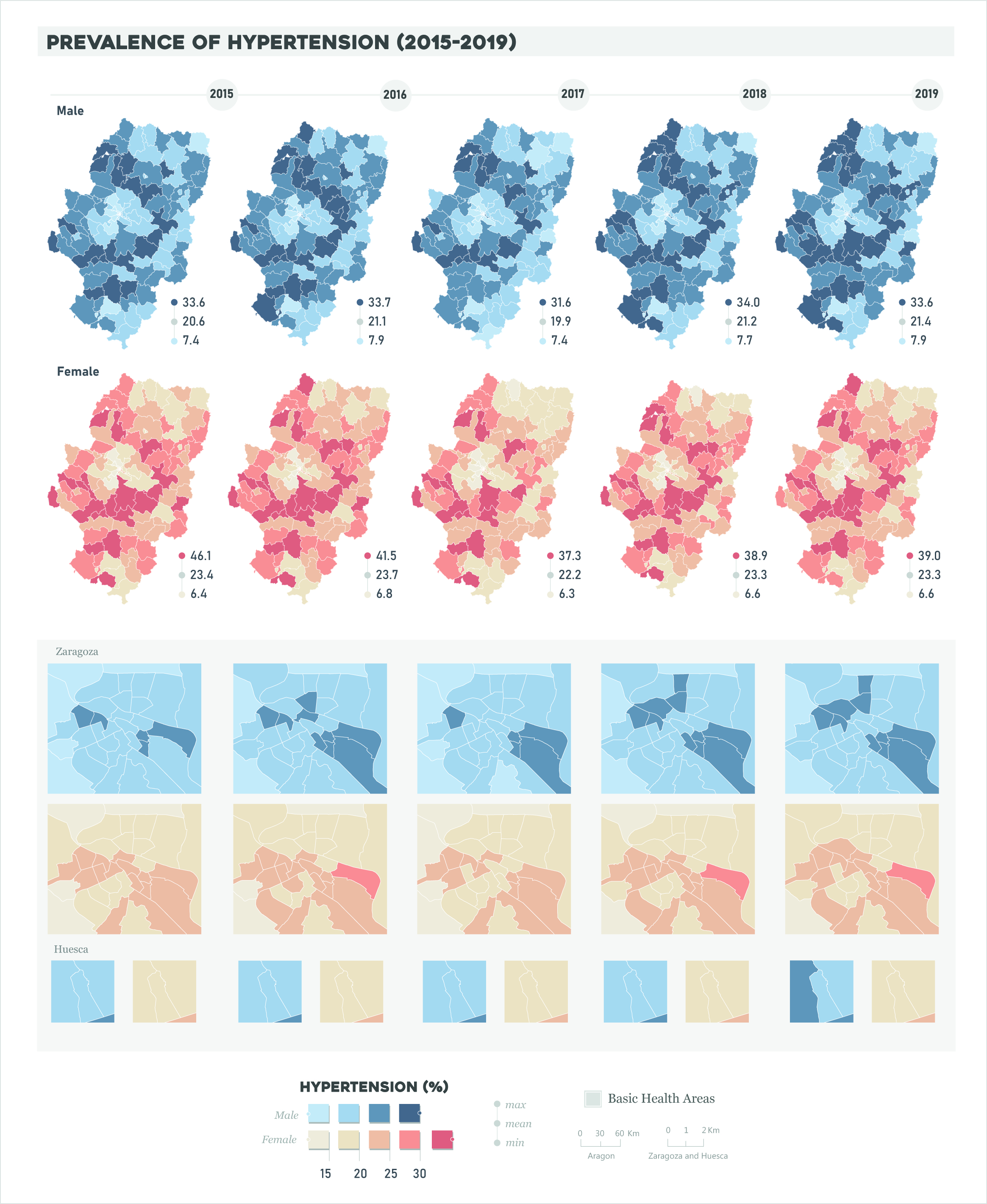

Supplement: Supplementary file 2 [file Image_2.JPEG]

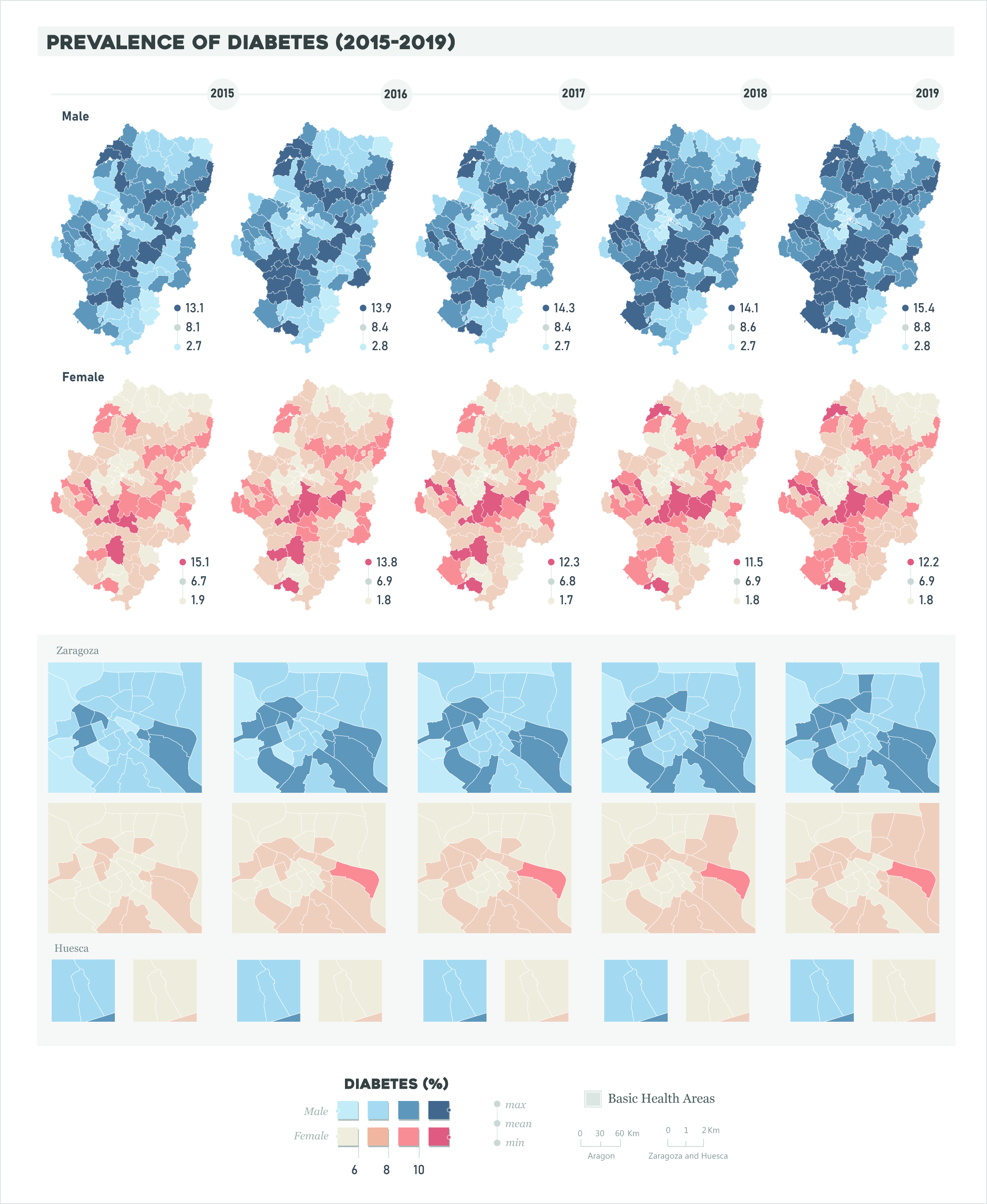

Supplement: Supplementary file 3 [file Image_3.JPEG]

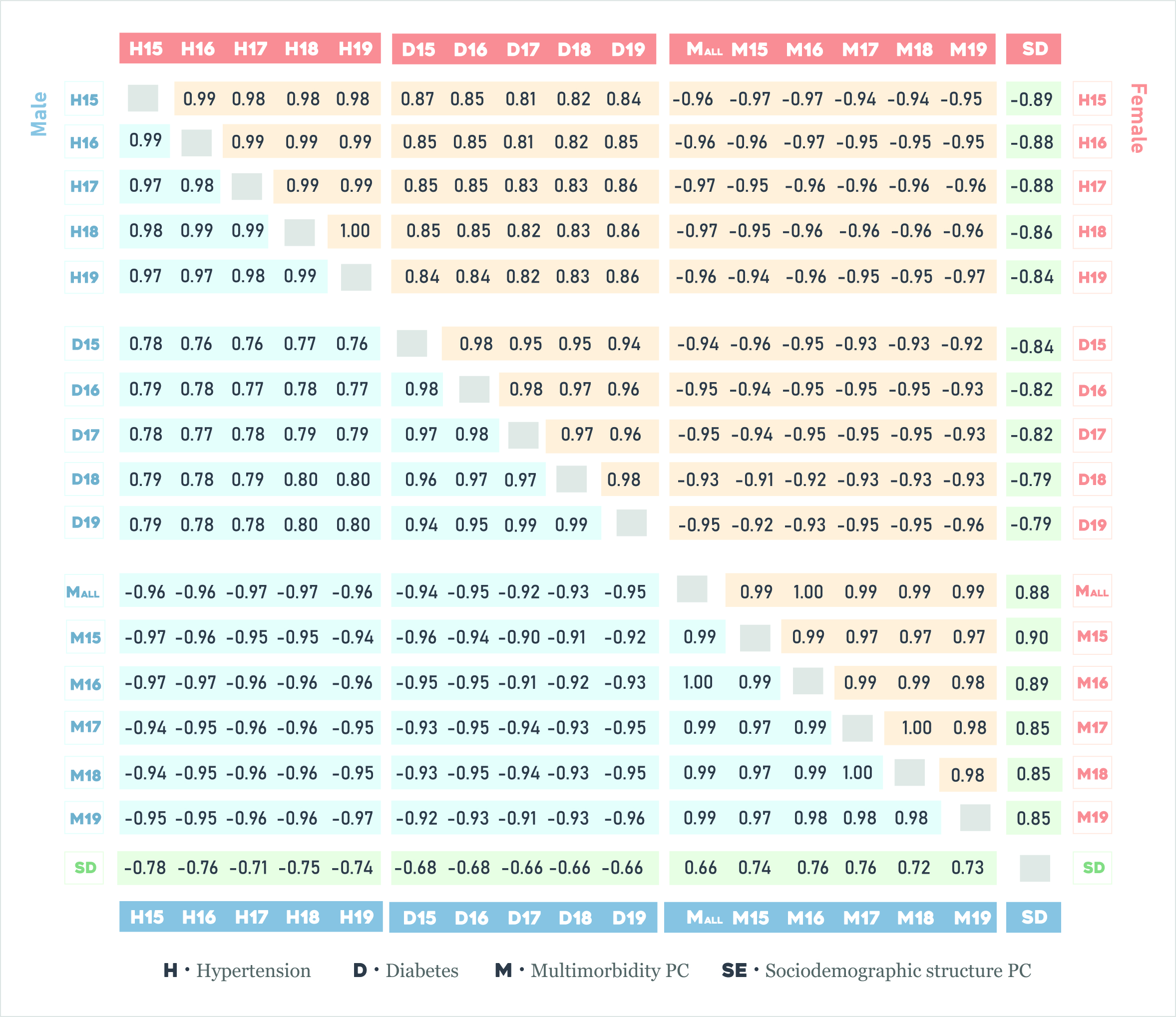

Supplement: Supplementary file 4 [file Image_4.JPEG]

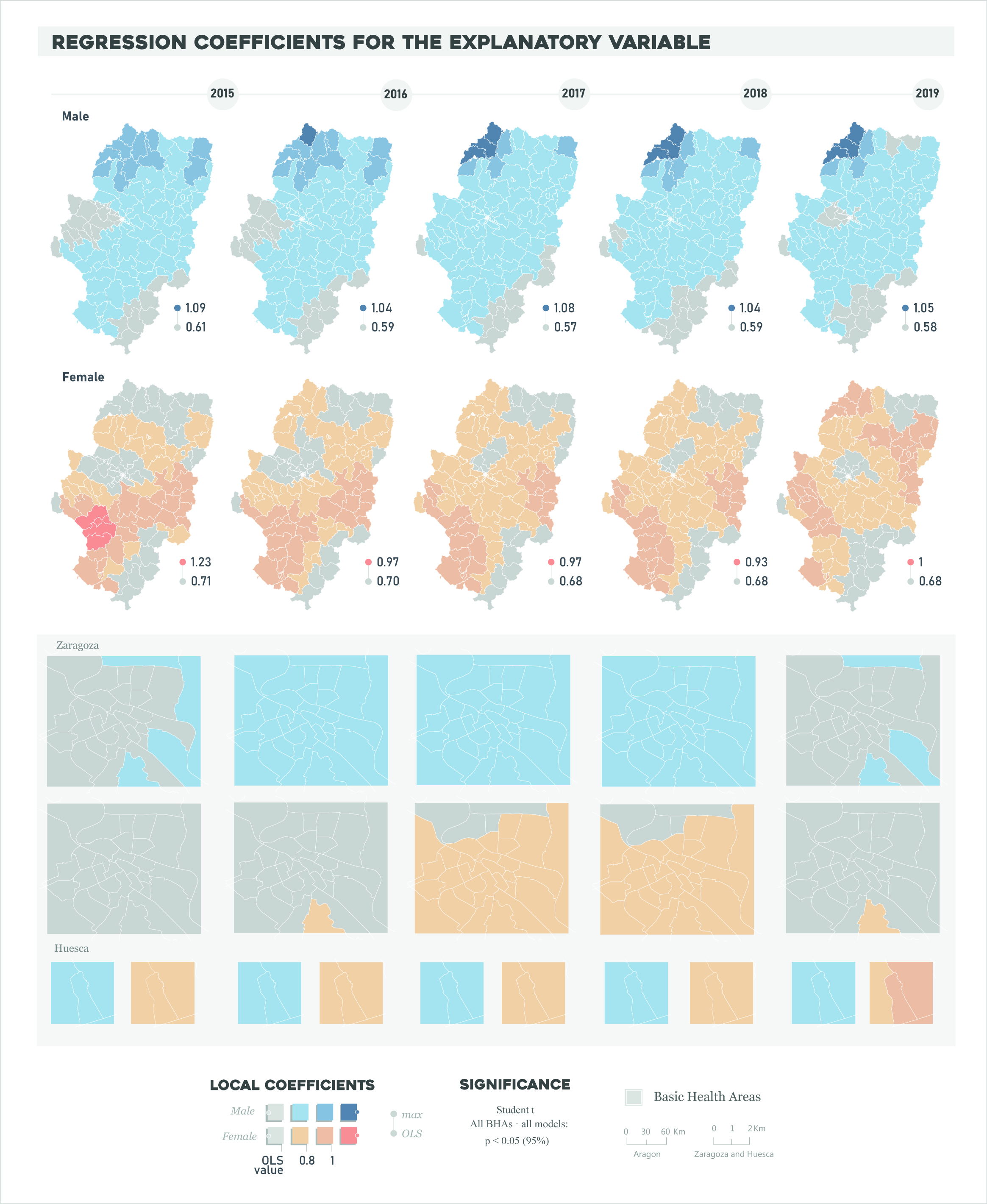

Supplement: Supplementary file 5 [file Image_5.JPEG]

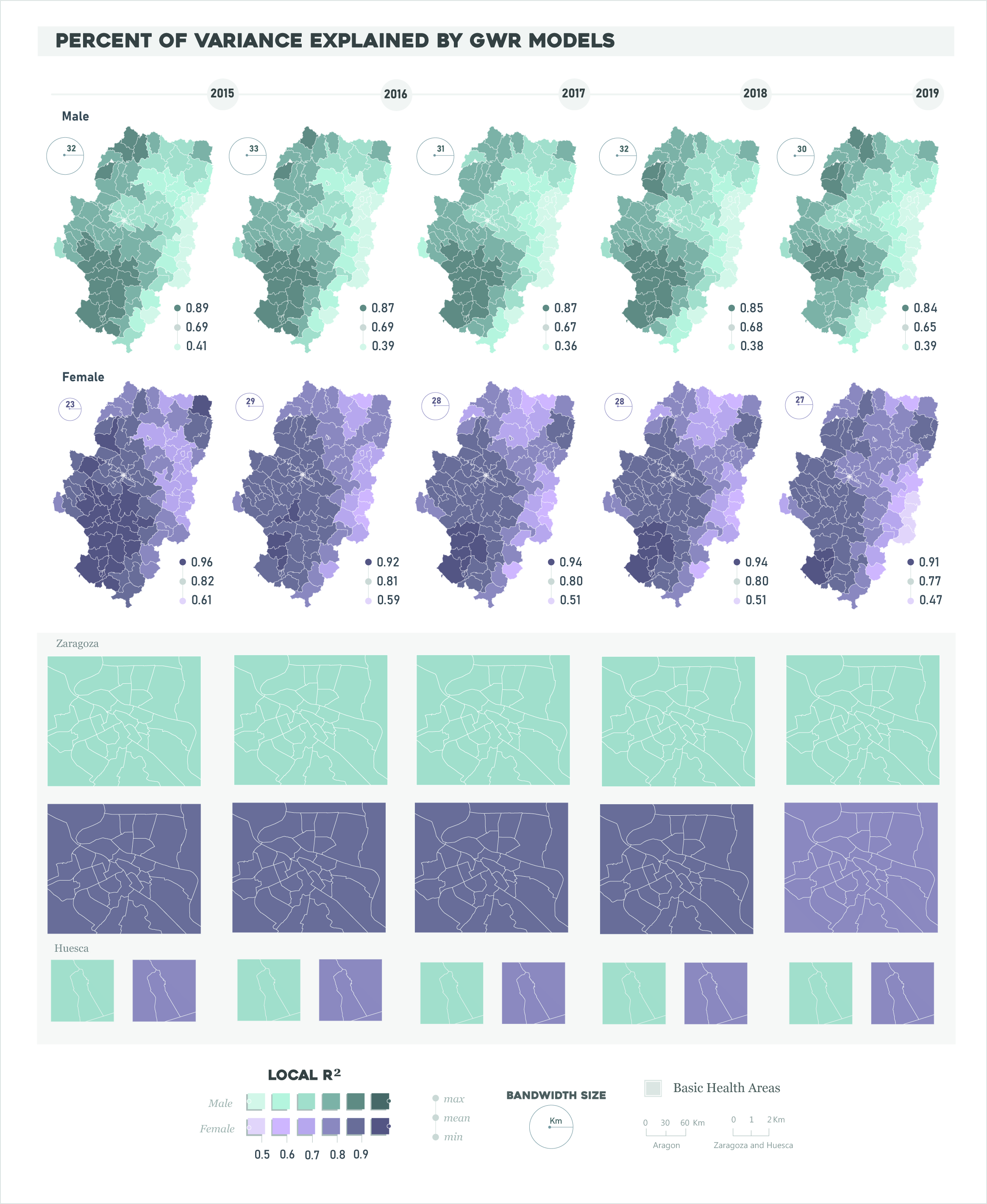

Supplement: Supplementary file 6 [file Image_6.JPEG]
